# Human wild-type full-length tau accumulation disrupts mitochondrial dynamics and the functions *via* increasing mitofusins

**DOI:** 10.1038/srep24756

**Published:** 2016-04-21

**Authors:** Xia-Chun Li, Yu Hu, Zhi-hao Wang, Yu Luo, Yao Zhang, Xiu-Ping Liu, Qiong Feng, Qun Wang, Keqiang Ye, Gong-Ping Liu, Jian-Zhi Wang

**Affiliations:** 1 Department of Pathophysiology, School of Basic Medicine and the Collaborative Innovation Center for Brain Science, Key Laboratory of Ministry of Education of China for Neurological Disorders, Tongji Medical College, Huazhong University of Science and Technology, Wuhan 430030, China; 2 Department of Physiology and Pathophysiology, Medical School, China Three Gorges University, Yichang 443002, China; 3 Institute of Pathophysiology, School of Basic Medical Sciences, Lanzhou University, Lanzhou 730000, China; 4 Endocrine Department, Liyuan Hospital, Tongji Medical College, Huazhong University of Science and Technology, Wuhan 430030, China; 5 Department of Pathology and Laboratory Medicine, Emory University School of Medicine, Atlanta, GA30322, USA; 6 Co-innovation Center of Neuroregeneration, Nantong University, Nantong 226001, China

## Abstract

Intracellular accumulation of tau protein is hallmark of sporadic Alzheimer’s disease (AD), however, the cellular mechanism whereby tau accumulation causes neurodegeneration is poorly understood. Here we report that overexpression of human wild-type full-length tau (termed htau) disrupted mitochondrial dynamics by enhancing fusion and induced their perinuclear accumulation in HEK293 cells and rat primary hippocampal neurons. The htau accumulation at later stage inhibited mitochondrial functions shown by the decreased ATP level, the ratio of ATP/ADP and complex I activity. Simultaneously, the cell viability was decreased with retraction of the cellular/neuronal processes. Further studies demonstrated that htau accumulation increased fusion proteins, including OPA1 and mitofusins (Mfn1, Mfn2) and reduced the ubiquitination of Mfn2. Downregulation of the mitofusins by shRNA to ~45% or ~52% of the control levels attenuated the htau-enhanced mitochondrial fusion and restored the functions, while downregulation of OPA1 to ~50% of the control level did not show rescue effects. Finally, abnormal mitochondrial accumulation and dysfunction were also observed in the brains of htau transgenic mice. Taken together, our data demonstrate that htau accumulation decreases cell viability and causes degeneration *via* enhancing mitofusin-associated mitochondrial fusion, which provides new insights into the molecular mechanisms underlying tauopathies.

Abnormal accumulation of wild-type human tau proteins is a hallmark of sporadic Alzheimer disease (AD)^1^. Expression of full-length human tau alone causes intracellular tau pathologies and behavioral deficits in mice^2^,^3^, while turning off tau expression attenuates the pathologies^4^. Reduction of endogenous tau also ameliorates memory deficits caused by β-amyloid (Aβ)^5^. These data suggest a crucial role of tau accumulation in neurodegeneration and memory loss. However, how intracellular accumulation of the wild-type tau impairs the cells’ function and eventually leads to neurodegeneration and memory impairments is currently not fully understood.

Mitochondrial dysfunction is an early pathological event of AD^6^,^7^, and abnormal mitochondrial morphology and distribution are detected in the postmortem AD brains and their fibroblasts^8^,^9^. Recent studies suggest that there is an intrinsic link between human tau and mitochondria. For instance, an N-terminal truncated tau (20–22kDa) is largely enriched in mitochondria of the AD brains and its amount in nerve terminal fields correlates with the pathological synaptic changes^10^. Mitochondrial dysfunction is also detected in P301L tau transgenic mice^11^. Deregulation of mitochondrial complex I with aging is tau-dependent^12^. Tau phosphorylation can antagonize cell apoptosis with the mechanisms involving Bcl-2 and caspase-3 in mitochondria^13^,^14^, while expression of fusion proteins attenuates apoptosis^15^. These observations suggest that intracellular accumulation of tau may cause neurodegeneration through disrupting mitochondrial functions, but the direct evidence for the role of wild-type full-length human tau in mitochondrial dynamic is still lacking.

Mitochondria, as dynamic organelles, regulate cell viability and morphology of the synapses^16^,^17^. The mitochondrial dynamics is regulated by continuous fusion and fission^18^,^19^. The fusion is regulated by mitofusins namely Mfn1 and Mfn2, the integral membrane proteins of the mitochondrial outer membrane^20^,^21^ and optic atrophy type 1 (OPA1), an inner mitochondrial membrane associated protein^15^,^22^. In yeast, exogenous expression of the mammalian homologues of Fis1 (hFis1 for human homologues) induces mitochondrial fragmentation, whereas in mammalian cells, dynamin like protein 1 (DLP1) plays a key role in mitochondrial fission and mitochondrial fission factor (Mff) functions as an essential factor for mitochondrial recruitment of DLP1 during fission^23^. The mitochondrial dynamics determines mitochondrial morphology, size, distribution and functions^24^. These observations suggest that intracellular accumulation of tau may antagonize acute apoptosis *via* modulating mitochondria fission/fusion and cause neurodegeneration through disrupting mitochondrial functions.

In the present study, we investigated whether and how htau is involved in mitochondria dysfunction and neurodegeneration. We show that overexpression of htau protein disrupts mitochondrial dynamics and the functions with a correlated reduction of cell viability and neurodegeneration, the mechanisms involve an enhanced fusion by an increased mitofusin accumulation.

## Results

### Intracellular tau accumulation enhances mitochondrial fusion without affecting their fission

**Figure 1:**
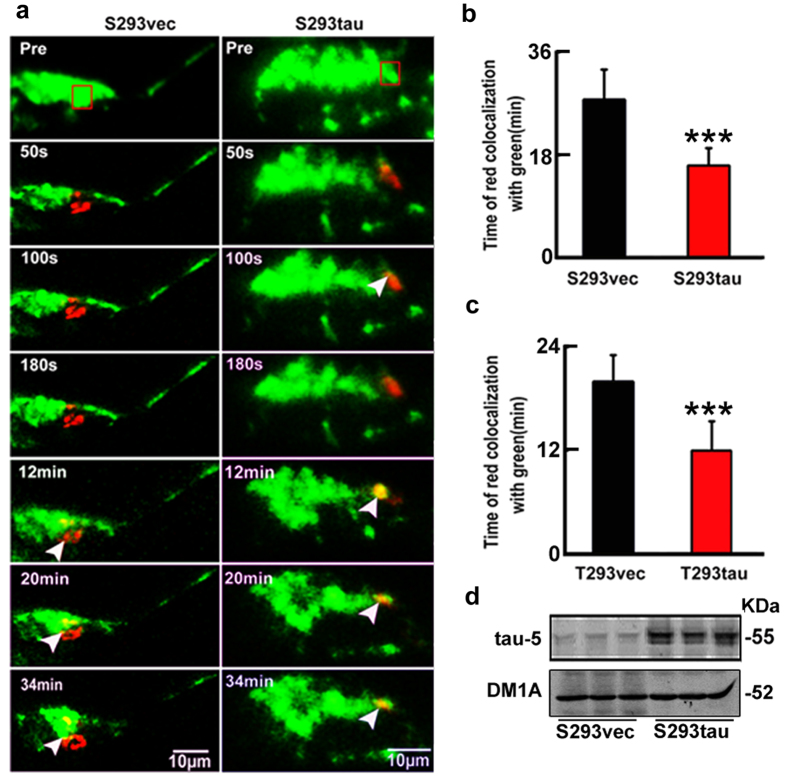
Expression of htau disrupts mitochondrial dynamics. (**a,b**) HEK293 cells with stable expression of htau (S293tau) or the vector (S293vec) were transfected with Mito-Dendra2 for 24 h. 50% power of 488 nm laser was applied for 50 s to the region of interest (ROI, square box) to allow full photo-conversion (from green to red) of all mitochondria within the ROI, then red mitochondria in ROI to turn yellow (arrowheads) in the entire cell was monitored and quantitatively analyzed using Zeiss-510 microscope. Data were presented as mean ± SD (n = 30, *p* = 0.000912). ***,*p* < 0.001. (**c**) HEK293 cells were transiently co-transfected with htau (T293tau) or the vector (T293vec) and Mito-Dendra2 (1 μg/ml, 1:1) for 24 h, and then red mitochondria in ROI to turn yellow was quantitatively analyzed. For quantification, the time used for a complete co-localization of red with green to form yellow was calculated. Data are presented as mean ± SD (n = 30, *p* = 0.00219). ***,*p* < 0.001. (**d**) Total tau levels in S293tau or S293vec cells were detected by Western blotting using antibody tau-5.

We have previously reported that intracellular accumulation of tau affects cell viability^13^. To explore the molecular mechanisms, we further investigated here the influence of tau on mitochondria, the vital organelle known for the essential role in energy metabolism and cell viability. We first measured mitochondrial dynamics in cells with stable expression of human tau or the vector (termed as S293tau and S293vec). A photo-convertible fluorescence protein, mito-Dendra2, was co-expressed to track mitochondrial dynamic in living cells. The cells show green signal under a low laser power (5%) at 24 h after expression of mito-Dendra2 (Fig. 1a). Then we selected several transfected cells with the similar morphology and applied higher laser power (50%) to a defined region of interest (ROI) in transfected cells to allow full photo-conversion of the mitochondria (from green to red). The fusion of a red mitochondrion with a green one generated a yellow mitochondrion. By measuring the percentage of red mitochondria that became yellow over time as an overall index for fusion events, we found that the average time for all red mitochondria in ROI to turn yellow is 27.6 ± 5.2 min in S293vec cells, whereas the average time was less than 16 ± 3.0 min in S293tau cells (Fig. 1a,b). The accelerated time for all red mitochondria in ROI to turn yellow was also observed in the cells with transient expression of htau (Fig. 1c). Since mitochondria fusion was affected by mitochondria mobility and velocity, we also assayed the mitochondria mobility and velocity in S293vec and S293tau cells. We found that the percent of moving mitochondria increased in S293tau cells and the retrograde movement of mitochondria in S293tau cells was higher than that of S293vec (Fig. S1). These data indicate that htau enhanced retrograde mitochondrial transport rate and fusion, which may explain the perinuclear mitochondrial accumulation.

### Overexpression of htau induces perinuclear accumulation of mitochondria

**Figure 2:**
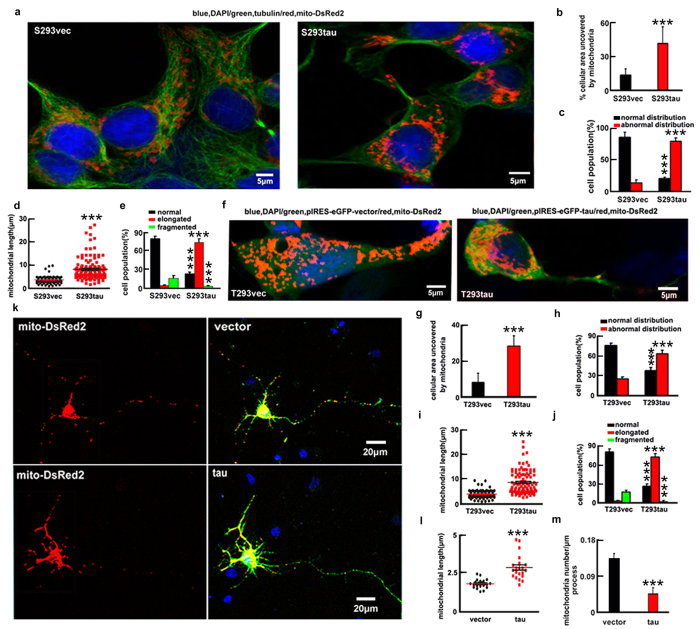
Expression of htau induces mitochondrial accumulation and elongation. (**a–e**) mito-DsRed2 was transfected into S293tau and S293vec cells respectively for 24 h, and then the cellular area devoid of mitochondria (**b**) (*p* < 0.0001), the cell population with normal or abnormal mitochondrial distribution (**c**) (*p*_normal_ < 0.0001, *p*_abnormal_ < 0.0001), the mitochondrial length (**d**) (*p* < 0.0001), and the cell population with different type of mitochondria (**e**) (*p*_normal_ < 0.0001, *p*_elongated_ < 0.0001, *p*_fragmented_ = 0.0038) were analyzed. (**f–j**) HEK293 cells were co-transfected with eGFP-labeled human tau or the vectors and mito-DsRed2 (T293tau or T293vec) for 24 h, and then analyzed as indicated. ((**g**) *p* < 0.0001; (**h**) *p*_normal_ < 0.0001, *p*_abnormal_ < 0.0001; (**i**) *p* < 0.0001; (**j**) *p*_normal_ < 0.0001, *p*_elongated_ < 0.0001, *p*_fragmented_ < 0.0001). (**k–m**) The rat primary hippocampal neurons (7 *div*) were co-transfected with eGFP-labeled human tau or the vector and mito-DsRed2 for 24 h, and then the mitochondrial length and number (counted in the neuronal processes 100 to 300 μm away from the cell body) were measured. The data were expressed as mean ± SD, at least 100 HEK293 cells or 60 primary neurons were counted in each group. ((**l**) *p* < 0.0001; (**m**) *p* < 0.0001). ***,*p* < 0.001 *vs* vec.

Then, we measured the cellular distribution and the morphologies of mitochondria (Fig. S2). In majority of S293vec cells (86.0 ± 8.0%), the mitochondria were uniformly distributed throughout the cytoplasm and only 12.5 ± 6.9% the cellular area was devoid of mitochondria. In contrast, in the majority of S293tau cells (79.8 ± 5.5%), the mitochondria were accumulated in the perinuclear area and the area devoid of mitochondria was increased to 39.9 ± 3.8% (Fig. 2a–c). As to the morphology, 73.9 ± 5.9% S293tau cells showed elongated mitochondria with an average length of 8.5 ± 5.0 μm, whereas 79.8 ± 4.1% S293vec cells exhibited short tubular structure with an average length of 3.6 ± 1.5 μm and only 4.0 ± 1.6% cells showed elongated mitochondria (Fig. 2d,e).

Apparent perinuclear accumulation of mitochondria with an increased length was also shown at 24 h after transient expression of htau (Fig. 2f–j). Microtubule stability was detected with no significant change of the acetylated α-tubulin level at 24 h after overexpression of htau, although an increased level of the acetylated α-tubulin was seen at 12 h (Fig. S3b), and no significant microtubule disruption was detected at 24 h (Fig. S3a), suggesting that the mitochondria impairment was independent of the microtubule disruption. Furthermore, the accumulation of mitochondria was also detected in rat primary hippocampal neurons cultured for 7 days *in vitro* (*div*) by co-expressing mito-DsRed2 and eGFP-tagged htau for 24 h (Fig. 2k–m). These data together strongly suggest that expression of htau enhances mitochondrial fusion and promotes their accumulation.

### Overexpression of htau impairs mitochondrial functions and causes neurodegeneration

**Figure 3:**
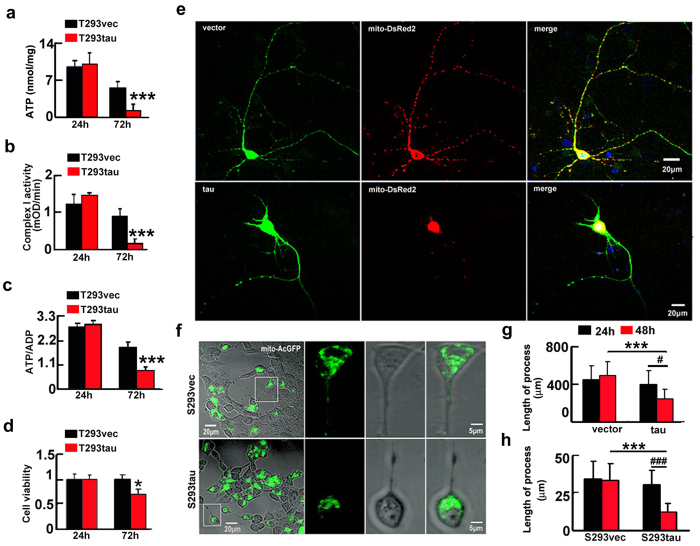
Expression of htau induces mitochondrial dysfunction with reduced cell viability and retraction of processes. (**a–d**) HEK293 cells were transiently transfected with htau (T293tau) or the vector (T293vec) for 24 and 72 h, and then the levels of ATP (**a**) (24 h, *p* = 0.789; 72 h, *p* = 0.00051), the activity of complex I (**b**) (24 h, *p* = 0.814; 72 h, *p* = 0.00011), the ratio of ATP/ADP (**c**) (24 h, *p* = 0.484; 72 h, *p* = 0.000041), and the cell viability (**d**) (24 h, *p* = 0.988; 72 h, *p* = 0.0228) were analyzed. The experiments were repeated at least three times with triplicates. Data were presented as mean ± SD. *,*p* < 0.05, ***,*p* < 0.001 *vs* vec. (**e–h**) Representative images show retraction/loss of cell processes and accumulation of mitochondria in rat primary hippocampal neurons co-transfected with htau and mito-DsRed2 for 48 h (**e**) and in S293tau cells co-transfected with htau and mito-AcGFP for 48 h (**f**), and quantification of the average length of the cell processes in primary neurons (**g**) or 293 cells (**h**). ((**h**) vec *vs* tau, *p*_24 h_ = 0.45, *p*_48 h_ < 0.0001; 24 h *vs* 48 h, *p*_vec_ = 0.540, *p*_tau_ = 0.0197. (**i**) S293vec *vs* S293tau, *p*_24 h_ = 0.398, *p*_48 h_ < 0.0001; 24 h *vs* 48 h, *p*_S293vec_ = 0.7967, *p*_S293tau_ < 0.0001). At least 100 HEK293 cells or 40 primary hippocampal neurons were counted each group. Data were expressed as mean ± SD, unpaired student *t* test. ***,*p* < 0.001, ^#^,*p* < 0.05, ^###^,*p* < 0.001.

We also observed that transient expression of htau decreased ATP level and the ratio of ATP/ADP, and as well as inhibited the complex I activity at 72 h (Fig. 3a–c), consequently, a remarkable reduction of cell viability was shown at 72 h (Fig. 3d). In the cells with stable expression of htau (S293tau) or the vector (S293vec), the ATP level, the ratio of ATP/ADP, the complex I activity, and the cell viability were all decreased at 72 h (Fig. S4a–d). These data confirm that overexpression of htau disrupts mitochondrial functions and reduces cell viability. Interestingly, an increased cell viability was detected at 48 h (Fig. S5), which supports our previous finding that overexpression of tau rendered the cells anti-apoptosis at early stage^13^. In rat primary hippocampal neurons cultured for 7 *div*, overexpression of htau for 48 h induced severe retraction or loss of neuronal processes (Fig. 3e,g). By time-lapse recording, retraction of the cell processes was shown after stable (Fig. 3f,h, and Supplementary video 1 and 2) or transient expression of htau for 48 h (Fig. S6). These data indicate that overexpression of htau causes neurodegeneration.

### Overexpression of htau increases fusion proteins and decreases the ubiquitination of Mfn2

**Figure 4:**
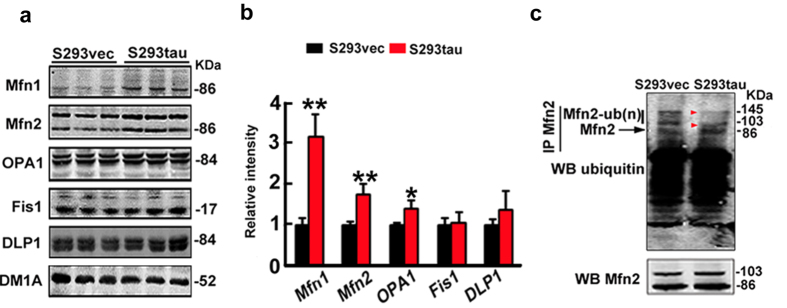
Expression of htau increases fusion proteins and reduces ubiquitination of Mfn2. (**a,b**) Levels of fusion proteins (Mfn1, Mfn2 and OPA1) or fission proteins (Fis1 and DLP1) in HEK293 cells with stable expression of htau (S293tau) or the vector (S293vec) cultured with serum deprivation for 24 h. DM1A against tubulin was used as a loading control. Data were expressed as mean  ±  SD. (Mfn1, *p* = 0.00125; Mfn2, *p* = 0.006; OPA1, *p* = 0.0102; Fis1, *p* = 0.88; DLP1, *p* = 0.2705). *,*p* < 0.05, **,*p* < 0.01 *vs* S293vec. (**c**) Immunoprecipitation by antibody against Mfn2 and Western blotting by anti-ubiquitin (upper panel) and Mfn2 (lower panel), respectively.

Mitochondrial morphology is regulated by dynamic fusion and fission; therefore, we measured the alteration of fusion proteins (Mfn1, Mfn2 and OPA1) and fission proteins (Fis1and DLP1). The results showed that fusion proteins (Mfn1, Mfn2, OPA1) significantly increased while the fission proteins (Fis1 and GLP1) were not changed much by stable expression of htau (Fig. 4a,b), suggesting that accumulation of fusion proteins may underlie the htau-enhanced mitochondrial fusion. We further measured the DLP1 level in the mitochondrial fraction, but no significant change was detected (Fig. S7). To explore the mechanisms underlying the htau-induced accumulation of the mitofusins, we measured the level of polyubiquitinated Mfn2 in the mitochondrial fraction. We found that expression of htau reduced the polyubiquitinated Mfn2 (Fig. 4c), while the ubiquitinated TOMM20 level had no significant change (Fig. S8), suggesting that an impaired ubiquitination may underlie the htau-induced Mfn accumulation.

### Downregulating mitofusins rescues the htau-induced mitochondrial impairments with restoration of cell viability

**Figure 5:**
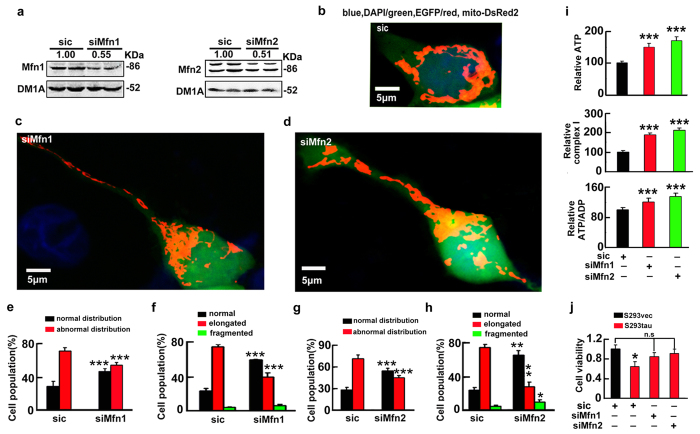
Knockdown of the mitofusins attenuates the htau-induced mitochondrial impairments with restoration of cell viability. (**a**) S293tau cells were co-transfected with mito-DsRed2 and shRNA of Mfn1 or Mfn2 for 24 h, and the knockdown effects were estimated by Western blotting, the scrambled siRNA (sic) was used as control. Each experiment was repeated at least three times. (**b–h**) Representative images and quantitative analyses shown the effects of Mfn1 (**c,e,f**), or Mfn2 (**d,g,h**) knockdown on mitochondrial morphology and the distribution in S293tau cells. For morphology analysis, at least 50 cells were counted in each group. ((**e**) *p*_normal_ = 0.0007, *p*_abnormal_ < 0.0001; (**f**) *p*_normal_ = 0.0004, *p*_elongated_ = 0.0004, *p*_fragmented_ = 0.094; (**g**) *p*_*normal*_ < 0.0001, *p*_abnormal_ < 0.0001; (**h**) *p*_normal_ = 0.003, *p*_elongated_ = 0.0027, *p*_fragmented_ = 0.0278). (**i,j**)The level of ATP, the ratio of ATP/ADP, and the activity of complex I analyzed at 48 h, and the cell viability analyzed at 72 h after knockdown of Mfns. ((**i**) ATP: siMfn1 *vs* siC, *p* = 2.79E-5; siMfn2 *vs* siC, *p* = 1.33E-6. Complex I: siMfn1 *vs* siC, *p* = 2.73E-6; siMfn2 *vs* siC, *p* = 2.92E-8. ATP/ADP: siMfn1 *vs* siC, *p* = 7.32E-5; siMfn2 *vs* siC, *p* = 3.07E-6. (**j**) S293vec+siC vs S293tau+siC, *p* = 0.0195. n = 6). Each experiment was repeated at least three times. Data were expressed as mean ± SD. *,*p* < 0.05, **,*p* < 0.01, ***,*p* < 0.001 *vs* siC.

To further verify the role of mitofusins in mediating the toxicity of htau accumulation, we used shRNA to knockdown the fusion proteins. We found that knockdown of Mfn1 or Mfn2 to ~45% or ~52% of the control levels attenuated the mitochondrial fusion and the accumulation (Fig. 5a–h), while downregulation of OPA1 to ~50% of the control level did not rescue the htau-induced mitochondrial impairment (Fig. S9). Simultaneously, knockdown of Mfn1/2 ameliorated the htau-induced mitochondrial dysfunction with restoration of cell viability at 72 h after tau overexpression (Fig. 5i,j). These data indicate that accumulation of the mitofusins mediates the htau-induced mitochondrial and cellular impairments.

### Mitofusin accumulation and mitochondrial dysfunction were detected in the brains of htau transgenic mice

**Figure 6:**
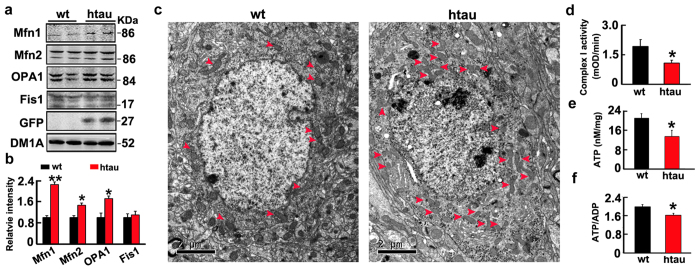
The htau transgenic mice showmitofusins accumulation with mitondrial dysfunction and their perinuclear accumulation. (**a,b**) The increased fusion proteins (Mfn1, Mfn2 and OPA1) in hippocampal extracts of htau transgenic mice (htau) compared with the age-matched wild-type littermates (wt) measured by Western blotting. (Mfn1, *p* = 0.0025; Mfn2, *p* = 0.0136; OPA1, *p* = 0.0142; Fis1, *p* = 0.798). (**c**) The perinuclear accumulation of mitochondria in hippocampal neurons measured by electron microscopy. (**d,f**) Mitochondrial dysfunction represented by the reduced levels of ATP (*p* = 0.0265), ratio of ATP/ADP (*p* = 0.0377), and complex I activity (*p* = 0.0452) in hippocampal extracts of the htau mice. Data were expressed as mean ± SD. *,*p* < 0.05, **,*p* < 0.01, ***,*p* < 0.001 *vs* wt.

Finally, we found that levels of Mfn1, Mfn2, and OPA1 were also significantly increased in hippocampus of 6 month-old htau transgenic mice (STOCK Mapttm1(EGFP)Klt Tg(MAPT)8cPdav/J) compared with the age-matched littermates (Fig. 6a,b), simultaneously, the perinuclear mitochondrial accumulation were also shown by electron microscopy and mitochondrial dysfunctions were found (Fig. 6c–f).

## Discussion

Intracellular accumulation of wild-type tau is the major cause of neurodegeneration in sporadic AD; however, the mechanism is not fully elucidated. As mitochondria homeostasis is vital for neuronal survival and death, we investigated the effects of htau on mitochondrial homeostasis and the molecular mechanisms. We found that intracellular htau accumulation, as seen in sporadic AD brains, resulted in an enhanced mitochondrial fusion and their perinuclear congestion with mitochondrial dysfunctions and neurodegeneration. The molecular mechanisms involve an impaired ubiquitination-associated increase of mitofusins, because simultaneous downregulation of the mitofusins rescued the htau-induced mitochondrial impairments and cell viability. Our findings provide new insights in the htau-induced neurodegeneration.

Upon overexpression of htau, apparent perinuclear accumulation of mitochondria was seen in both cultured cells and the htau transgenic mice. Simultaneously, we observed an enhanced retrograde axonal transport of mitochondria in the htau-expressing cells, though unexpected, these data can perfectly explain the perinuclear accumulation of the mitochondria as observed in our current study. The mitochondria fusion is dependent on increased motility and fluctuations in microtubule post translational modifications^25^. We find that overexpression of htau increases Mfn1 and Mfn2, which not only promote mitochondria fusion but also interacts with Miro/Milton complex during mitochondrial transport^26^; Knockdown of Miro2 in cultured neurons induces transport deficits identical to loss of Mfn2^26^, therefore silence Miro/Milton may also rescue the htau-induced mitochondrial fusion. Additionally, we have previously proposed that tau may play a dual role in leading the cell escape acute apoptosis and triggering the chronic neurodegeneration^27^,^28^,^29^, based on our systemic studies^13^,^14^,^30^,^31^,^32^. We believe that the perinuclear accumulation of mitochondria observed in the current study may represent an early compensatory response of tau to preserve the cell bodies from the acute apoptosis, while the chronic neurodegeneration is the unavoidable fate of the cells with increasing tau accumulation and depriving mitochondria from the axon terminals.

Mitochondrial function is regulated by dynamic fusion and fission. Previous studies indicate that mitochondrial fusion is neuron protective, leading to the exchange of mitochondrial DNA, reorganization of mitochondrial cristae, and protect cells from apoptosis, whereas mitochondrial fission seems a sign of apoptosis^33^,^34^. Here, we show that expression of htau promotes mitochondrial fusion with increased cell viability at 48 h after tau transfection, which is consistent with our recent reports showing that expression of htau renders the cells more resistant to the acute cell apoptosis induced by exogenous apoptotic inducers^13^,^14^,^30^,^31^,^32^. We also observed that mitochondria elongation and their perinuclear cumulating appeared at 24 h, when the neurite density and the morphology were not changed much; while degeneration of the neuronal processes and loss of neurites were seen at 48 h after overexpression of htau. The sequential appearance of mitochondrial abnormalities and the axon degeneration upon expression of htau were also detected by live cell time-lapse imaging, which strongly supports that the abnormal mitochondria dynamics is upstream of neurodegeneration. Together with our previous studies^13^,^14^,^30^,^31^,^32^, we propose that tau accumulation ameliorates apoptosis at early stage while it causes neurodegenerasis (degenerative cell death) at later stage, because the mitochondrial dysfunction leads to the deficits of basic energy supply for cells to survival. Therefore, the current study provides evidence supporting the dual role of tau in AD neurodegeneration^27^,^28^,^29^.

A previous study in drosophila and fibroblast showed that overexpression of human R406W mutant tau could elongate mitochondria with the mechanisms involving reduced fission^35^. Here, we also detected mitochondrial elongation with an enhanced fusion but without change of fission after overexpression of wild-type htau. Another study reported that levels of the mitofusins decreased in the hippocampus of the AD patients, and β-amyloid treatment reduced the mitofusin level in primary hippocampal neurons^8^,^9^. In the current study, we found a remarkable elevation of the mitofusins, which was positively correlated with abnormal tau accumulation *in vitro* and in htau transgenic mice. These data indicate that β-amyloid and different types of tau proteins may impair mitochondrial dynamics and induce mitochondrial dysfunction with different mechanisms. Additionally, a previous study indicated that overexpression of wild type tau in primary cortical neurons suppressed mitochondrial motility and fusion and induced mitochondrial fragmentation^25^. The exact reason for the discrepancy is currently not clear, but we think different sources of the primary neurons, i.e., the hippocampus *versus* cerebral cortex, may be one of them.

How the intracellular htau accumulation may cause elevation of the mitofusins? Since we did not see any significant change of the mRNA levels of the mitofusins, we speculate that the htau accumulation may affect the proteolytic turnover of the fusion proteins. This point was supported by the significantly reduced ubiquitination of Mfn2, a crucial step for the proteolysis, in the htau-expressing cells. The mitofusin ubiquitination is regulated by PTEN-induced kinase 1 (PINK1)-parkin pathway^36^,^37^,^38^,^39^, and the mitochondrial residing of PINK1/parkin is essential for the proteasome-dependent proteolysis of the mitofusins^38^,^39^. It is conceivable that tau accumulation may reduce the recruitment of PINK1/parkin into the injured mitochondria and thus decrease the ubiquitination and proteolysis of the mitofusins, and eventually lead to mitochondrial fusion and formed elongated mitochondria. These findings reveal new molecular targets that mediate tau toxicities.

## Conclusion

Taken together, we find in the present study that intracellular accumulation of htau results in an enhanced mitochondrial fusion with mitochondrial dysfunction and neurodegeneration. The mechanisms involve an impaired ubiquitination-associated elevation of the mitofusins.

## Methods

### Immunofluorescence and live cell time-lapse imaging

For immunofluorescence, cells were cultured on 12-well plates. After various treatments, the cells were fixed for 40 min with 4% paraformaldehyde in PBS (pH 7.4) and permeabilized for 10 min at room temperature in PBS containing 0.5% Triton X-100. Cells were blocked with 5% bovine serum albumin (BSA) (A9418, Sigma) for 40 min, and further incubated with primary antibody at 4 °C overnight, and then incubated for 1 h at 37 °C with Rhodamine Red-X- (Molecular Probes, R6393, Eugene) or Oregon Green 488-conjugated secondary antibodies (Molecular Probes, O6383, Eugene). For the triple labeling studies, DAPI (0.1 μg/ml, D9542, Sigma) was used for the nuclear staining. All fluorescence images were captured with a Zeiss LSM 710 laser-scanning confocal fluorescence microscope (Zeiss, Jena, Germany) equipped with Zen software (Zeiss) and a 63 × 1.4 NA oil-immersion objective lens (the expected lateral resolution for 488 nm excitation light is about 178 nm per pixel). Red images of mito-DsRed2 were collected using 543 nm excitation and a 560 nm filter; and green fluorescence images were collected using 488 nm excitation and a 500–550 nm filterwith the pinhole set to 1 Airy Unit. Image analysis was performed with Image-Pro Plus 6.0 software (Media Cybernetics, CA, USA).

The measurement of neurite and axon length was performed using NIH ImageJ software as described previously^40^. The mean length was calculated by measuring the total number of pixels in the images from which the pixels of the cell bodies had been subtracted and divided by the average pixel number per micrometer of the axons and the total number of cell bodies in the image (total length per neuron = total number of pixels (only axons) × (pixel number per micrometer)^−1 ^× (number of neurons)^−1^). The fluorescence intensity of immunostaining was calculated by ImageJ software.

For live cell time-lapse imaging, the cells were transfected with mito-Dendra2 for 24 h, and then put in a well-equipped live imaging station (Zeiss CTI-Controller 3700) with controlled CO_2_, humidity and temperature. Images were captured with a Zeiss LSM 510 inverted laser scanning confocal fluorescence microscope (Zeiss, Jena, Germany) equipped with Zen software (Zeiss) and EC Plan Neofluor 40×/0.75 M27 objective lens (the expected lateral resolution for 488 nm excitation light is 332 nm per pixel). Images of red signal were collected using 543 nm excitation with a 560 nm filter with pinhole of 706 μm, and green fluorescence was collected using 488 nm excitation with a 500–550 nm filter with pinhole of 640 μm and Z plane distance of 500 nm. Fast and sufficient photoswitch of mito-Dendra2 was achieved by exposing highly zoomed area to 50% power of 488 nm laser for 20 iterations. During time-lapse imaging, frames were captured every 10 s for at least 1 h without apparent phototoxicity or photobleaching. Image analysis was also performed with open source image-analysis programs WCIF ImageJ (developed by W. Rasband) and Image-Pro plus 6.0 (Media Cybernetics). The average velocities (μm/s) of moving mitochondria were quantified using the ImageJ Manual Tracking plugin. Because mobility was usually accompanied by brief pauses, along with changes in direction, mitochondria that paused for >5 s or moved <2 μm throughout the movie were omitted. Mitochondrial mobility was assessed by counting the number of mitochondria moving as a percentage of the total number of mitochondria. Mitochondria in a field of view were manually classed as moving if they moved >2 μm throughout the movie. Oscillating mitochondria were identified as moving back and forth no more than 2 μm from their initial position. Immobile mitochondria were manually identified as not moving and not oscillating.

### Measurement of mitochondria distribution and the morphology

For mitochondrial distribution assay, the mitochondria were labeled by mito-DsRed2 and the total cellular area was visualized by tubulin staining. For the calculation, a broken line was drawn along bordering mitochondria to encircle an area containing all mitochondria within the cell. The size of the perinuclear area outside this circled area was measured (i.e., perinuclear area devoid of mitochondria) and its ratio to total perinuclear area (circled by full line) was calculated and presented as a percentage (Fig. S2). Abnormal mitochondria distribution was defined as >10% of the cytoplasmic area devoid of mitochondrion. For mitochondrial morphology assay, the length longer than 5 μm was defined as mitochondrial elongation, while the length shorter than 2.5 μm was defined as mitochondrial fragmentation (Fig. S2b). In primary neurons, the cell body and neuronal process 100 to 300 from the body were used for measuring average mitochondrial length, and the neuronal processes 100 to 300 μm away from the cell body were used for measuring mitochondrial number by using Image-Pro plus 6.0.

### Statistical analysis

All data were collected and analyzed in a blinded manner. Data were expressed as mean ± SD and analyzed using SPSS 10.0 statistical software (SPSS Inc. Chicago, IL, USA). P-values were calculated with unpaired Student’s t-test (two-tailed, type 2) for one-way comparisons and with ANOVA followed by post hoc test (Student’s t-test, two-tailed, type 2) for multiple comparisons.

## Additional Information

**How to cite this article**: Li, X.-C. *et al.* Human wild-type full-length tau accumulation disrupts mitochondrial dynamics and the functions *via* increasing mitofusins. *Sci. Rep.* **6**, 24756; doi: 10.1038/srep24756 (2016).

## Supplementary Material

Supplementary Information

Supplementary Video 1

Supplementary Video 2
