# Supplementary material for: Human wild-type full-length tau accumulation disrupts mitochondrial dynamics and the functions via increasing mitofusins
Source: Sci Rep. 2016 Apr 21;6:24756. doi: 10.1038/srep24756 (PMC4838862; doi:10.1038/srep24756)
Supplement: Supplementary Information [file srep24756-s1.pdf]

Supplementary data

Human wild-type full-length tau accumulation disrupts mitochondrial dynamics and the functions *via* increasing mitofusins

Xia-Chun Li, Yu Hu, Zhi-hao Wang, Yu Luo, Yao Zhang, Xiu-Ping Liu, Qiong Feng, Qun Wang, Keqiang Ye, Gong-Ping Liu, Jian-Zhi Wang

SUPPLEMENTARY INFORMATION

Supplementary Methods

Plasmids, antibodies and reagents

Mito-DsRed2 and mito-Dendra2 plasmids were gifts of Dr. Xiongwei Zhu (Case Western Reserve University, Cleveland, OH); human pIRES-eGFP-Tau40 plasmid was a gift of Dr. Khalid Iqbal (New York State Institute for Basic Research in Developmental Disabilities, Staten Island, NY). The PCI-neo-Tau40 was constructed in our lab.The RNA interference vector was generated *via* the pcDNA 6.2-GW/EmGFP-miR construct (Invitrogen). The siRNA sequences targeting the open reading frame region of human mitochondrial fusion proteins were as follows: Mfn1, TTTAGCGACTAAACACATCAG, Mfn2, GGAAGACATTGAGTTCCAT, OPA1, GCTGAACGCAGTATTGTTA.

Mouse monoclonal antibody (mAb) anti-OPA1(#612607) was from BD Bioscience; rabbit polyclonal antibody (pAb) anti-Fis1 (#IMG-5113A) was from IMGENEX; mAb anti-Mfn1 (sc-166644) was from

Santa Cruz Biotechnology; mAb anti-GAPDH (mAbcam 9484), pAb anti-ubiquitin(ab14372) and anti-Mfn2 (#ab50843) were from Abcam; mAb anti-α-tubulin (T9026) was from Sigma; Lipofectamine2000 (11668-019) was from Invitrogen; NeuroFECT^{TM} (T800075) was from Genlantis. ATP assay kit was from Biovison, Complex I enzyme activity microplate assay kit was from Abcam, ADP/ATP ratio assay kit was from BioAssay Systems, Cell counting Kit-8 (CCK8) was from Dojindo Molecular Technologies; and the assays were carried out by following the manufacturer's instructions.

Cell culture

The human embryonic kidney293 (HEK293) were grown in Dulbecco's Modified Eagle's medium (DMEM medium) (12491-015, Gibco), supplemented with 10% (v/v) fetal bovine serum and 1% penicillin/streptomycin, in a humid 5% CO_2 incubator at 37°C. After growing 24 h in plates or flasks, the cells were transfected with the indicated plasmid(s) using Lipofectamine2000 according to the manufacturer's instructions. To establish the cell line with stable expression of htau40, we used culture medium containing 200 μg/ml geneticin for the selection.

For primary neuron cultures, 18 days embryonic (E18) rat hippocampus were seeded at 30,000-40,000 cells per well on 6-well plates coated with Poly-D-Lysine/Laminin (354455BD, Bioscience) in neurobasal medium (21103-049, Invitrogen) supplemented with 2% B27/0.5 mM glutamine/25 mM glutamate. Half the culture medium was changed every 3 days with neurobasal medium supplemented with 2% B27 and 0.5 mM glutamine. All cultures were kept at 37ºC in a humidified 5% CO_2 containing atmosphere. More than 90% of the cells were neurons after they were cultured for 7 to 17 *div*; this was verified by positive staining for the neuronal specific markers microtubule-associated

protein-2 (MAP2, dendritic marker, AB5622, Millipore). At 7 to 10 *div*, neurons were transfected with tau plasmids and mito-DsRed2 2:1 using NeuroFECT™ according to the manufacturer's protocol.

Co-immunoprecipitation

For co-immunoprecipitation (IP), cells were harvested in IP buffer (50 mM Tris-HCl (pH 7.4), 150 mM NaCl, 1% NP-40, 10% glycerol, 10 mM NaF, 1 mM Na_3VO_4, 100 mg/ml PMSF and complete mini protease inhibitor cocktail). Cell lysates were rotated at 4°C for 1 h and the supernatants were obtained by centrifugation at 17,500×g for 15 min at 4°C. Soluble fractions were combined with 50 µl Protein G-Dynabeads (100.04D, Invitrogen) pre-incubated with anti-Mfn2 followed by overnight rotation at 4°C. Dynabeads complexes were washed 3 times with IP buffer supplemented with 500 mM NaCl. Immunoprecipitates were eluted by heating at 95°C for 5 min in sample buffer containing 5% 2-mercaptoethanol before Western blotting.

Western blotting

For Western blotting, equal amounts of protein were separated by 10% sodium dodecyl sulfate-polyacrylamide gel electrophoresis (SDS-PAGE) and transferred onto nitrocellulose membranes. The membranes were blocked in 5% non-fat milk for 1 h at room temperature and then incubated with primary antibody at 4°C overnight. Then the blots were incubated with IRDye 800CW-conjugated affinity-purified anti-mouse IgG (610-132-121, Rockland) and IRDye 800CW anti-rabbit IgG secondary antibody (611-132-002, Rockland) for 1 h at room temperature. Immunoreactive bands were visualized using the Odyssey Infrared Imaging System (Licor Biosciences, Lincoln, NE, USA). All the animal methods were carried out in accordance with the "Policies on the

Use of Animals and Humans in Neuroscience Research" revised and approved by the Society for Neuroscience in 1995. All experimental protocols were approved by the Institutional Ethics Committee of Tongji Medical College, Huazhong University of Science and Technology.

Electron microscopy

Sections for electron microscopy were prepared as previously described[1]. In brief, rats were anesthetized and perfused with normal saline followed by 0.5% glutaraldehyde in 4% paraformaldehyde. The brain was removed and placed in 2.5% glutaraldehyde for 12 h at 4 ºC. The CA3 region was removed from the brain slices. After 30 min in 100 mM phosphate buffer containing 1% OsO4, the samples were dehydrated in ethanol and embedded in Epon 812. Finally, thin sections, cut with a diamond knife on an ultramicrotome (UCT, Leica, Germany), were collected on mesh grids coated with a thin Formvar film, and were stained in uranyl acetate and lead citrate for 25 min. The images were viewed using an electron microscope (FEI Tecnai G2 12, Holland).

Reference

1. Yang, Y. et al. EPAC null mutation impairslearning and social interactions via aberrant regulation of miR-124 and Zif268translation. *Neuron* **73** (4), 774-788 (2012).

SUPPLEMENTARY FIGURES

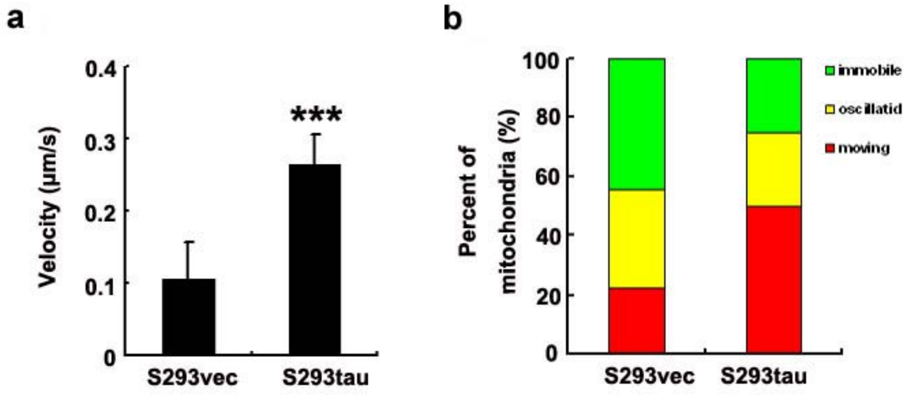

Supplementary Figure 1. The velocity of moving mitochondria and the mobility of mitochondria of S293vec and S293tau cells.

(a) The velocity of moving mitochondria of S293vec and S293tau cells (p=0.000184). ***, $p<0.001$ *vs* S293vec. (b) The mobility of mitochondria of S293vec and S293tau cells.

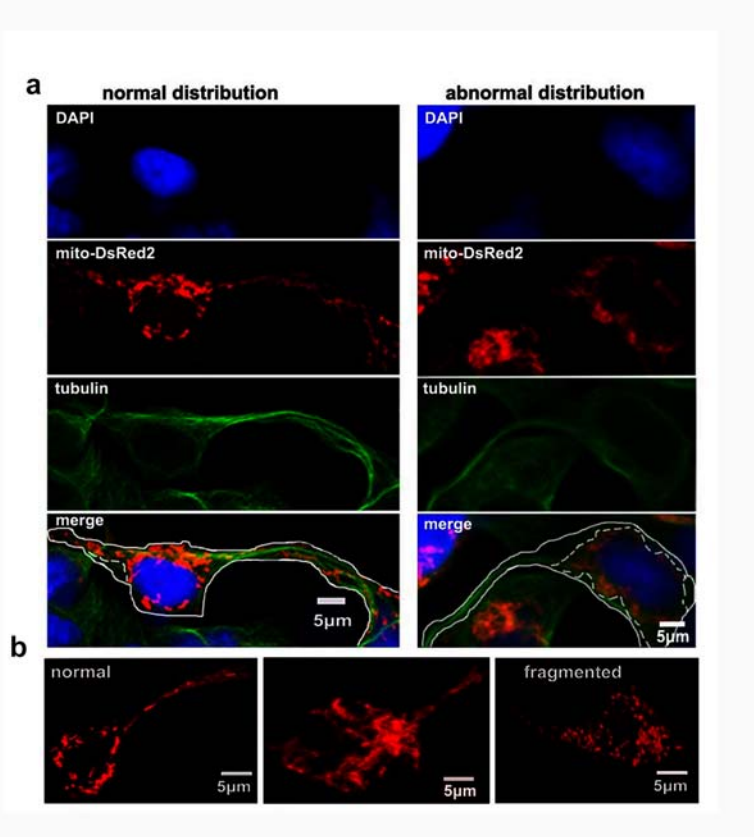

Supplementary Figure 2. The representative images of mitochondrial distribution and morphology in wild-type HEK293 cells, and the criteria for the analyses.

(a) The size of the somatodendritic area outside the dashed line circled area (i.e., somatodendritic area devoid of mitochondria) and its ratio to total somatodendritic area (circled by solid line) were calculated and presented as a percentage. The abnormal mitochondrial distribution was defined as >10% of the cytoplasmic area devoid of mitochondrion. (b) Normal mitochondria were defined as 2.5~5 μm; the length longer than 5 μm was defined as mitochondrial elongation, while the length shorter than 2.5 μm was defined as fragmentation.

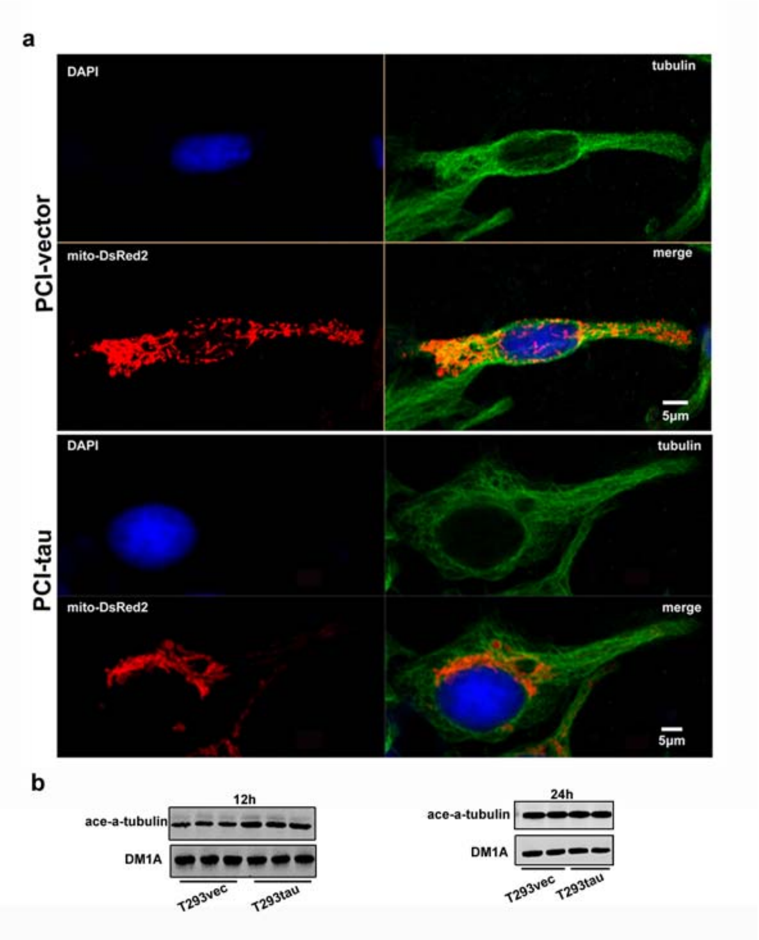

Supplementary Figure 3. Expression of htau for 24 h does not disrupt microtubule network.

(a) Immunostaining of HEK293 cells with tubulin antibody demonstrated that expression of htau (PCI-tau) for 24 h did not significantly affect the microtubule network (green, tubulin), although mitochondrial accumulation (red, mito-DsRed2) was already shown compared with the vector (PCI-vector). DAPI (blue) was used to stain the nuclei. (b) The levels of acetylated-α-tubulin were detected at 12 or 24 h after tau overexpression.

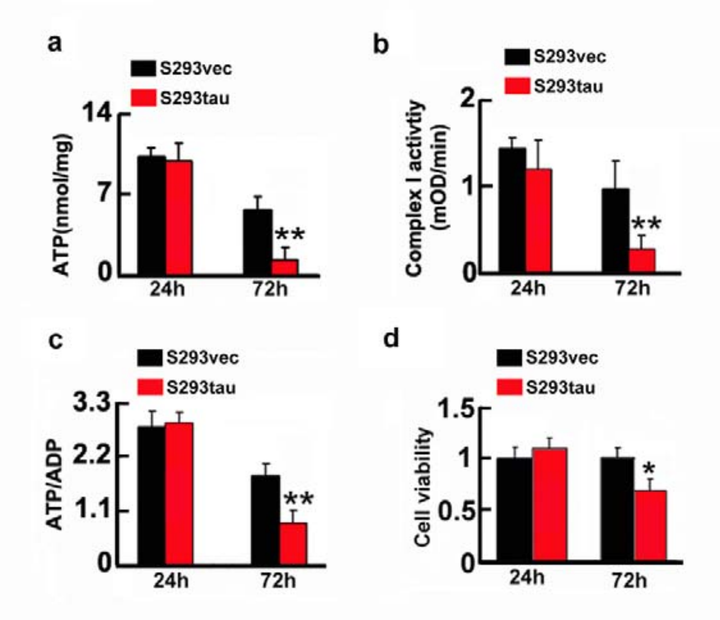

Supplementary Figure 4. Stable expression of htau induces mitochondrial dysfunction with reduced cell viability.

HEK293 cells with stable expression of human tau (S293tau) were starved for 24 h and 72 h, and then the levels of ATP (a) (p_{24h}=0.616, $p_{72\mathrm{h}}$=0.000051), the activity of complex I (b) (p_{24h}=0.118, $p_{72\mathrm{h}}$=0.00013), (c) the ratio of ATP/ADP(p_{24h}=0.623, $p_{72\mathrm{h}}$=0.000053), and (d) cell viability (p_{24h} =0.311, $p_{72\mathrm{h}}$=0.023) were measured. The experiments were repeated at least three times with triplicates. Data were expressed as mean±SD, unpaired student's t test. *, p<0.05, **, p<0.01 *vs* vec.

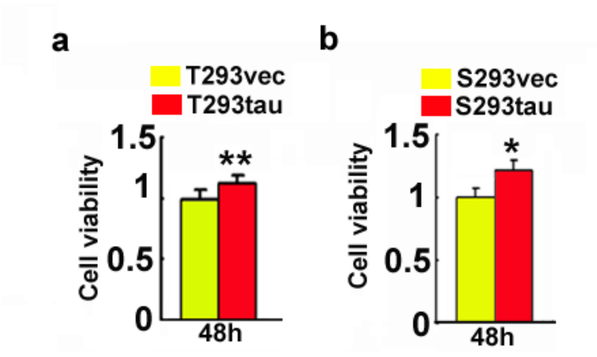

Supplementary Figure 5. Tau overexpression attenuated cell apoptosis.

HEK293 cells were transiently transfected with htau (T293tau, a) or stable htau (S293tau, b) for 48 h, and then the cell viability (a, p=0.0013; b, p=0.0455) were analyzed. The experiments were repeated at least three times with triplicates. Data were presented as mean ±SD. *, p<0.05, **, p<0.01 *vs* vec.

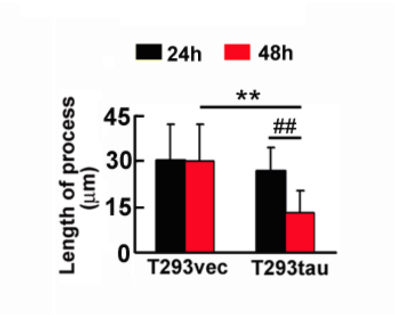

Supplementary Figure 6. Transient expression of htau causes retraction of cell processes.

The HEK293 cells were co-transfected with htau or the vector and mito-DsRed2 for 24 and 48 h, and then the length of the cell processes was analyzed. The data were expressed as mean±SD, (T293vec vs T293tau, p_{24h}=0.1023, p_{48h}<0.0001; 24 h vs 48 h, $p_{T293vec}$=0.952, $p_{T293tau}$<0.0001. at least 50 cells were counted in each group. **, p<0.01; ##, p<0.01.

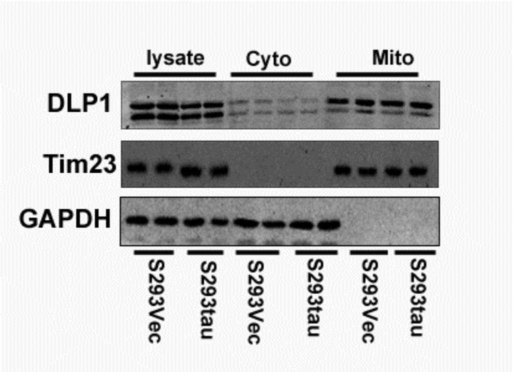

Supplementary Figure 7. Tau had no effect in the level of DLP1 in the mitochondrial fraction.

Levels of DLP1 in the cell lysate, cytoplasmic (Cyto) and mitochondrial (Mito) fractions of HEK293 cells with stable expression of htau (S293tau) or the vector (S293vec) were detected by Western blotting. GAPDH is a marker of cytoplasmic proteins, while Tim23 is the marker of mitochondrial proteins.

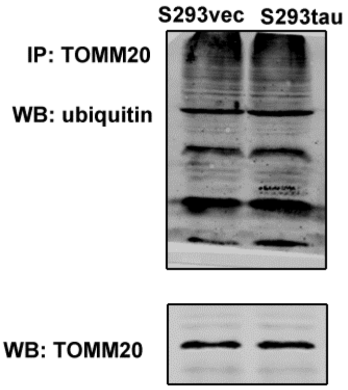

Supplementary Figure 8. Ubiquitinated TOMM20 level had no significant change between tau overexpression and the vector control.

Immunoprecipitation by antibody against TOMM20 and western blotting by anti-ubiquitin and TOMM20, respectively.

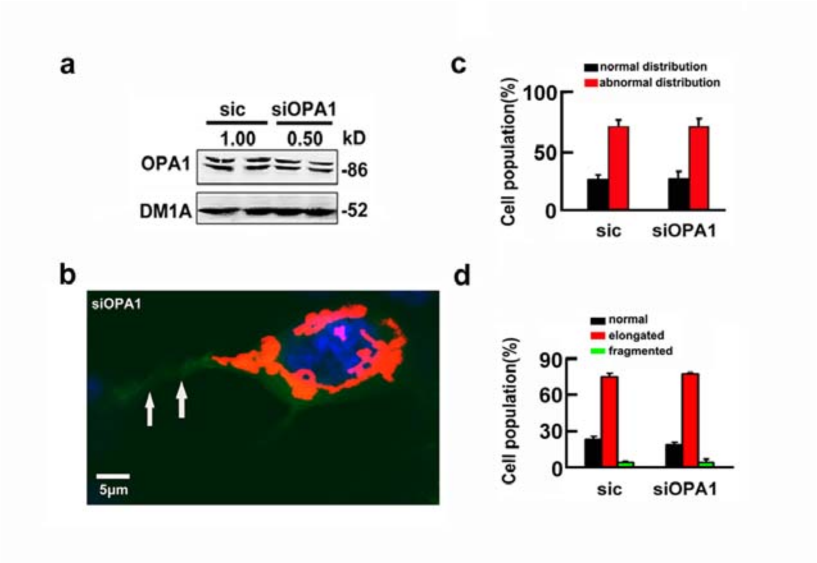

Supplementary Figure 9. Knockdown of OPA1 does not affect the htau-induced mitochondria abnormal distribution and fusion.

(a) HEK293 cells with stable expression of htau (S293tau) were co-transfected with mito-DsRed2 and shRNA of OPA1 (GFP) for 24 h, and then OPA1 protein level was detected by Western Blotting. (b) Representative images, (c) the cell population with normal or abnormal mitochondrial distribution, and (d) the cell population with different type of mitochondria were analyzed. At least 60 cells were counted in each group. Data were expressed as mean±SD. (siOPA *vs* siC, c, p_{normal}=0.775, p_{abnormal} =0.791; d, p_{normal}=0.6917, $p_{\text{elongated}}$=0.198, $p_{\text{fragmented}}$=0.972)

Supplementary video 1. Vector has no effect in cell processes of HEK293 cells.

HEK293 cells were transfected with Mito-DsRed2 for 23 h followed by transfection of pIRES-EGFP (vector), then frames were captured every 120 s for 60 min at 23 h and 48 h with Zeiss 510 to observe the alterations of mitochondrial dynamics and cell processes. The movie was made by collecting the video data at 23 h and 48 h and played for 12 s. It was shown that mitochondrial keeps fission/fusion balance without cell process retraction after HEK293 cells transfected with pIRES-EGFP for 48 h.

Supplementary video 2. Tau induces cell processes retraction.

HEK293 cells were transfected with Mito-DsRed2 for 23 h followed by transfection of human pIRES-EGFP-Tau40 (tau), then frames were captured every 120 s for 60 min at 23 h and 48 h with Zeiss 510 to observe the alterations of mitochondrial dynamics and cell processes. The movie was made by collecting the video data at 23 h and 48 h and played for 12 s. The mitochondria cumulating were shown when htau was expressed for 24 h, and retraction of the neuritis was observed at 48 h.
